# N-3 polyunsaturated fatty acids restore Th17 and Treg balance in collagen antibody-induced arthritis

**DOI:** 10.1371/journal.pone.0194331

**Published:** 2018-03-15

**Authors:** Ji Young Kim, Kyu Lim, Kyung Hee Kim, Jin Hyun Kim, Jin Sun Choi, Seung-Cheol Shim

**Affiliations:** 1 Division of Rheumatology, Daejeon Rheumatoid & Degenerative Arthritis Center, Chungnam National University Hospital, Daejeon, Republic of Korea; 2 Department of Biochemistry and Cancer Research Institute, College of Medicine, Chungnam National University, Daejeon, Republic of Korea; 3 Department of Pathology, Cancer Research Institute, College of Medicine, Chungnam National University, Daejeon, Republic of Korea; University of South Florida St Petersburg, UNITED STATES

## Abstract

N-3 polyunsaturated fatty acids (PUFA) have anti-inflammatory effects and were considered useful for the treatment of rheumatoid arthritis (RA). Recently, several studies suggested that n-3 PUFAs attenuated arthritis in animal model and human, however the mechanism is still unclear. Interleukin 17 (IL-17) is a pro-inflammatory cytokine mainly produced by T helper 17 (Th17) cells which cause tissue inflammation and bone erosion leading to joint destruction. In contrast, regulatory T (Treg) cells down-regulate various immune responses by suppression of naïve T cells. The imbalance between Th17 cells and Tregs cell is important for the pathogenesis of RA. Here, we investigated whether n-3 PUFAs attenuate arthritis in collagen antibody-induced arthritis (CAIA) model. We used fat-1 transgenic mice expressing the Caenorhabditis elegans fat-1 gene encoding an n-3 fatty acid desaturase that converts n-6 to n-3 fatty acids, leading to abundant n-3 fatty acids without the need of a dietary n-3 supply. Clinical arthritis score was significantly attenuated in fat-1 mice compared to wild type (WT) mice on day 7 (1.6±1.8, p = 0.012) and day 9 (1.5±1.6, p = 0.003). Ankle thickness also decreased significantly in fat-1 mice compared to WT mice (1.82±0.11, p = 0.008). The pathologic finding showed that inflammatory cell infiltration and bone destruction were reduced in fat-1 mice compared to WT. The expression levels of IL-17 and related cytokines including IL-6 and IL-23 decreased in the spleen and ankle joint tissue of fat-1 mice compared to WT mice. Furthermore, Treg cells were expanded in the spleen of fat-1 mice and Treg cell differentiation was significantly higher in fat-1 mice than in wild type (p = 0.038). These data suggest that n-3 PUFAs could attenuate arthritis through increasing the expression of FoxP3 and the differentiation of Treg, while reducing IL-17 production. Therefore, dietary supplementation of n-3 PUFAs could have a therapeutic potential for the treatment of RA.

## Introduction

Rheumatoid arthritis (RA) is an autoimmune disease characterized by chronic inflammation of a joint synovium, leading to progressive bone and cartilage destruction [1]. Recently, T helper 17 (Th17) cells have been suggested to play key roles in RA induction and maintenance. Th17 cells are pro-inflammatory cells, which produce interleukin 17 (IL-17) and differentiation is dependent on IL-6, IL-23 and TGF-β [2,3]. IL-17 is raised in synovial fluids of RA patients and in inflamed joints of experimental induced arthritis models. IL-17 induces inflammatory cell infiltration into the synovium and tissue inflammation, leading into bone resorption and erosion [4]. In contrast CD4^+^CD25^+^Foxp3^+^ regulatory T cell (Treg) regulates the immune response to keep peripheral self-tolerance [5]. Th17 and Treg cells antagonize each other’s function, the imbalance between Th17 and Treg cells may be important in the development of RA.

N-3 polyunsaturated fatty acid (n-3 PUFA) has shown anti-inflammatory effect through immune cell inhibition [6–8]. N-3 PUFAs are primarily obtained from the diet, only low conversion of α-linolenic acid (ALA) to eicosapentanoic acid (EPA) and docosahexaenoic acid (DHA) [9]. Recently, experimental animal studies reported that n-3 PUFAs suppress inflammatory cytokine synthesis [10]. In addition, n-3 PUFAs reduced the number and differentiation of pro-inflammatory Th17 cells [7–8, 11] and increased number of Treg cells in vivo [12]. A fat-1 transgenic mouse expresses the Caenorhabditis elegans fat-1 gene encoding an n-3 fatty acid desaturase that converts n-6 to n-3 fatty acids (which is absent in mammals), leading to abundant n-3 fatty acids with reduced levels of n-6 fatty acids in their organs and tissues, without the need of a dietary n-3 supply [13]. Furthermore, fat-1 mice delayed the development of osteoarthritis [14] and decreased the severity of arthritis in K/BxN serum transfer arthritis model [15]. Compared with n-3 PUFAs feeding studies, studies using fat-1 mice could withdraw confounding factors of diet such as nutrient composition, total caloric intake, duration of feeding and contamination of trace elements. The fat-1 mice have got resistibility in various inflammatory disease models such as pancreatitis [16] and asthma [17].

We used the collagen antibody-induced arthritis (CAIA) model which is induced by the administration of cocktail of monoclonal antibodies recognizing conserved epitopes located within the CB11 fragment of collagen. CAIA offers several advantages, including rapid disease onset, high uptake rate, and the capacity to use genetically modified mice, such as transgenics and knockouts [18].

The purpose of our study was to investigate whether n-3 PUFAs in fat-1 mice attenuated CAIA.

## Materials and methods

### Animals

#### Ethics Statement

All animal experiments were performed under the Institutional Animal Care and Use Committee of Chungnam National University Hospital (IACUC, Approval No. CNUH-015-A0010) which arrpoved the current study protocol and the ARRIVE Guidelines (Animals in Research: Reporting In Vivo Experiments). All efforts were made for minimize animal suffering. The animals were kept in groups (5 animals per cage) under controlled conditions with 12 hour light/dark cycle and monitored once daily for nine days following the first immunization. In this study, anaerobes were not necessary because they did not perform surgical surgery to induce arthritis. Also, this was not necessary for pain relievers because it was not a drug treatment experiment, and arthritis were milder and duration of experiments was short. At the end of the survival experiment, live animals were euthanized by zoletil(90mg/kg)/rompun(30mg/kg) overdose. Total 11 animals were used for this study, all the mice were euthanized.

#### Fat-1 transgenic mice

Fat-1 transgenic mice were kindly provided from Dr.J.X. Kang at the Harvard Medical School (Boston, MA, USA) [19]. The mice were kept under specific pathogen free conditions in standard cage, and 7–8 weeks old males were used for the experiment.

#### Collagen antibody-induced arthritis mice

To induce arthritis, the AthritoMab^TM^CllmAb cocktail (4 mg/mouse, MD bioscience GmbH, Zurich, Switzerland) was injected intravenously. Three days after antibody administration, 100 μg LPS (*Escherichria coli* 055:B5; MD Biosciences) was injected intra-peritoneally. The experiment was ended 9 days after antibody administration.

### Arthritis evaluation

The severity of arthritis was assessed by clinical arthritis score and hind paw thickness. Clinical arthritis scores were evaluated using a scale of 0–4 for each limb (0 = no swelling; 1 = slight swelling and erythema; 2 = moderate swelling and erythema; 3 = severe swelling and erythema; and 4 = maximal inflammation with joint rigidity). The maximum possible score for each mouse was 16. Ankle thickness was measured using a caliper placed across the ankle joint at the widest point. On day 9, the mice were sacrificed and joint tissues were harvested from each animal for end point histology.

### Determination of cytokine concentrations

IL-6, IL-17 and IL-23 levels in whole blood were determined using Milliplex® MAP kit (KomaBiotech, Korea) according to the manufacturer’s instruction.

BioRad (Hercules, CA) Bio-Plex kits were used for cytokine assays from joint lysates. All samples were run in duplicate and were assayed for murine IL-6, IL-17 and IL-23 multi-plex bead based kits. The assays were run according to manufactures recommended procedures. In brief, all buffers and diluents were warmed to room temperature prior to use. Lyophilized cytokine standards were reconstituted first to a mater standard stock using 250ul diluted according to manufacturer’s direction. Four-fold serial dilutions of the master standard stock provided eight concentrations used to determine a standard curve. The concentration ranges of the standards were: IL-6: 7.8–8000 pg/ml; IL-17: 39–40000 pg/ml; IL-23: 342–350000 pg/ml. After completion of all steps in the assay, the plates were read in Luminex 200 system and the data analyzed using BioPlex Manager 6 software.

### Immunoblotting

Protein was isolated from joint tissue using RIPA buffer. After sonication, the joint lysate was collected by centrifugation at the speed of 13000g at 4°C for 10 min to remove lysate debris and stored in aliquots at -80°C until used. The protein concentration in the cell extracts were determined by the Bio-Rad protein assay (Bio-Rad, CA). Fifty micrograms of tissue protein were subjected to SDS-PAGE on 4% to 20% tris-glycin gels for IL-6, IL-17, IL-23 and actin. The separated proteins were electrophoretically transferred onto the nitrocellulose membrane (Bio-Rad). Nonspecific binding was blocked with 0.5% tween20 in TBS (TBS-T) contain 5% nonfat milk for 1h at room temperature. The membranes were then incubated overnight at 4°C with individual primary antibodies then incubated with the HRP conjugated secondary antibodies at 1:10000 dilution in TBS-T. The protein bands were visualized with the enhanced chemiluminescence westernblotting detection system.

### RNA isolation and real-time Q-PCR

RNA was isolated from spleen tissue using Trizol reagent (Invitrogen, Carlsbad, CA) and then precipitated with isopropanol and dissolved in RNA storage buffer (Ambion, Austin, TX). First strand cDNA was synthesized by ReverTra Ace qPCR RT Master Mix with gDNA Remover kit (Toyobo, Osaka, Japan) following manufacturers’ instruction. Specific primers for each gene were designed using primer3 software. Q-PCR was performed with primers as shown in Table 1.

**Table 1:** Sequences and accession numbers for primers used in RT-PCR.

Gene	sequence	NCBI accession no.
GAPDH	For: CGTCCCGTAGACAAAATGGT	NM_008084
	Rev: TTGATGGCAACAATCTCCAC	
IL-6	For: AGTTGCCTTCTTGGGACTGA	NM_031168
	Rev: TCCACGATTTCCCAGAGAAC	
IL-23	For: CAGGGAACAAGATGCTGGAT	NM_031252
	Rev: GGCTAGCATGCAGAGATTCC	
IL-17	For: TCTCTGATGCTGTTGCTGCT	NM_010552
	Rev: CGTGGAACGGTTGAGGTAGT	
TNF-α	For: GCTGTCCCTGCGCTTCA	NM_013693 [14]
	Rev: CTCGTCCCCAATGACATCCT	
FoxP3	For: CCTATGGCTCCTTCCTTGGC	NM_001199348
	Rev: ATGAAGTGTGGTCTGTCCTGG	

Amplification of 1ul of cDNA was performed by SYBR mixture using iQ^TM^Supermix PCR Master Mix (Bio-Rad Laboratories, Hercules, CA, USA). The PCR program used an initial step of 95^°^C for 3 min for pre-denaturation, followed by denaturation at 95^°^C, annealing and extension at 58°C using the CFX connector (Bio-Rad Laboratories, Hercules, CA, USA). Quantization of relative gene expression was calculated by the comparative Ct method (2^−ΔΔCt^) as described by the manufacturer. Data were normalized to GAPDH mRNA levels. Three independent experiments were carried out to study mRNA levels.

### Splenocyte CD4+ T cells isolation and culture

The removed spleen was immediately transfered to 50ml tube containing PBS. For the cell isolation, the spleen was ground using a cell strainer (100 μm, nylon) and lymphocytes were separated using a Ficoll-Hypaque gradient. Splenic CD4^+^ T cells were isolated using the CD4^+^ T cells isolation kit from Miltenyi Biotec (Miltenyi Biotec) and cultured in RPMI1640 media. For differentiation to Treg cells, splenic CD4^+^ T cells were activated with anti-CD3 (0.5μg/ml) and CD28 (1μg/ml) antibodies and treated with anti TGF-β (5μg/ml) antibody for 3 days.

### Flow cytometry analysis (FACS)

For Treg cell analysis, cells were permeabilized with fixation/permeabilzation buffer (eBioscience, San Diego, CA) and stained with Mouse Regulatory T cell Staining kit#2 (eBioscience, San Diego, CA) according to the manufacturer’s instruction. Briefly, after cell surface staining with anti-CD4-FITC and anti-CD25-PE for 30min, to block mouse Fc receptors, cell was incubated with CD16/CD32 for 15min. Then, followed by incubation with fixation/permeabilization buffer for 30 min and staining with anti-Foxp3-APC for 30min.

### Histology and immunohistochemistry

On day 9, the mice were euthanized and the hind ankle joints were removed. Mouse joint tissues were fixed with 10% formalin, decalcified for 3 days in 10% EDTA and embedded in paraffin wax blocks. Five μm sections were stained with hematoxylin-eosin. For immune-histochemical staining, anti-IL-6 (Santa Cruz Biotechnology, Inc., Santa Cruz, CA), IL-17(Santa Cruz Biotechnology, Inc., Santa Cruz, CA) andIL-23 (Abcam Ltd., Cambridge, UK) antibodies were used. To eliminate endogenous peroxidase activity, sections were treated with 3% H_2_O_2_ (Sigma, St. Louis, MO). Sections were subsequently treated with 10% normal serum (BD) and antibody labeling was detected using DakoREAL^TM^ EnVision^TM^ Detection systems, Peroxidase/DAB+, Rabbit/mouse (DAKO Corporation, Carpinteria, CA). The staining of joint tissue was determined semi-quantitatively using a four grade scale from 0 (little or no stain) to 4 (large number of positive cells).

### Statistical analysis

All values in the results were expressed as mean ± SD. The significant differences between groups were determined using non-parametric statistic method, two tailed Mann-Whitney U test was used (PASW, Ver. 18.0. Chicago, IL). A value of *p*< 0.05 was considered statistically significant.

## Results

### Collagen antibody-induced arthritis was attenuated in the fat-1 mice

**Fig 1:**
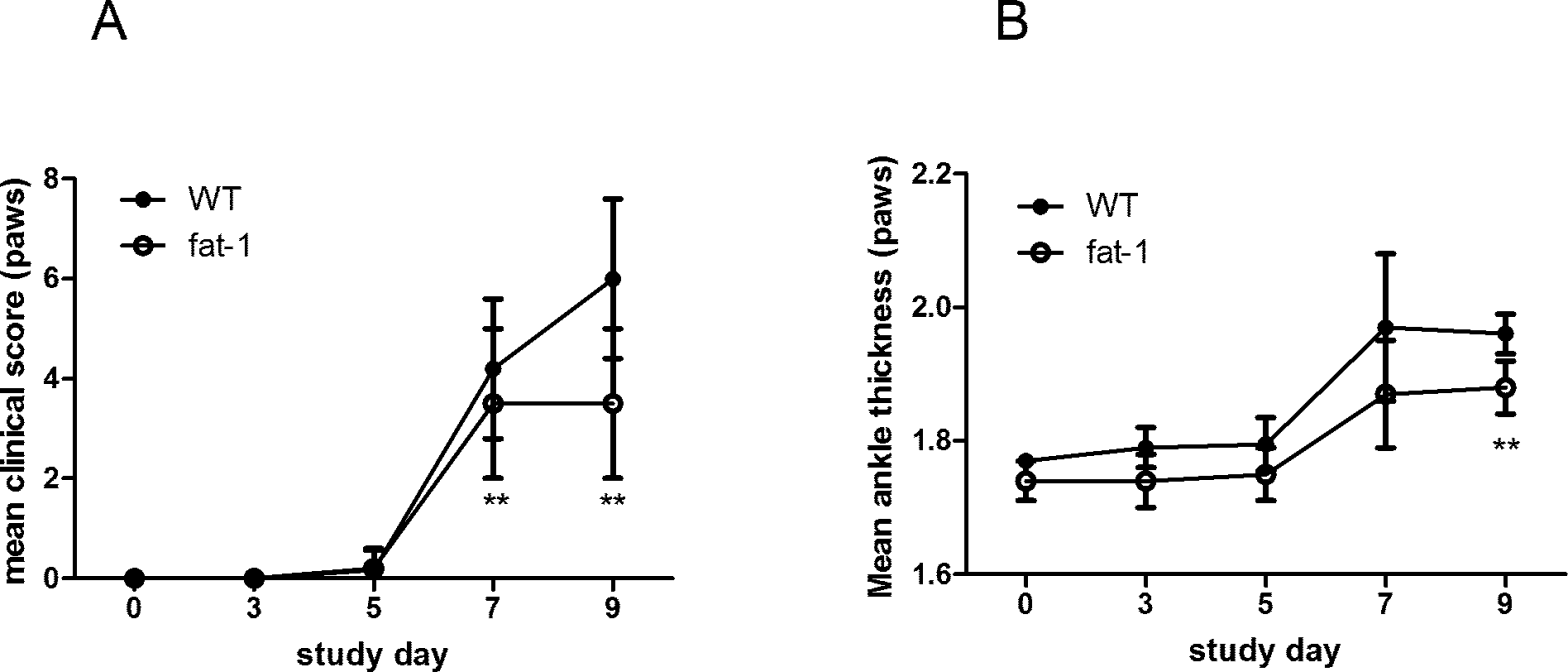
Clinical disease scores and ankle thickness in the WT and fat-1 mice in which arthritis was induced by anti-collagen antibodies. Arthritis was induced with 4mg of collagen antibodies in the WT and fat-1 mice. Clinical arthritis scores (A) and ankle thickness (B) were monitored every other day until day 9. Values are the mean±SD (n = 5 mice/group). (*p<0.05, **p<0.01) WT: wild type. Statistical significance test was done by Mann-Whitney U-test.

To determine whether n-3 PUFAs affected joint inflammation in collagen antibody-induced arthritis, male C57/B6 wild type (WT) mice and fat-1 transgenic mice were immunized with anti-collagen antibodies and monitored signs of arthritis until day 9. WT mice developed severe ankle swelling which was maximized on day 9. However, fat-1 transgenic mice showed significantly milder ankle swelling compared to WT mice (Fig 1A and 1B).

**Fig 2:**
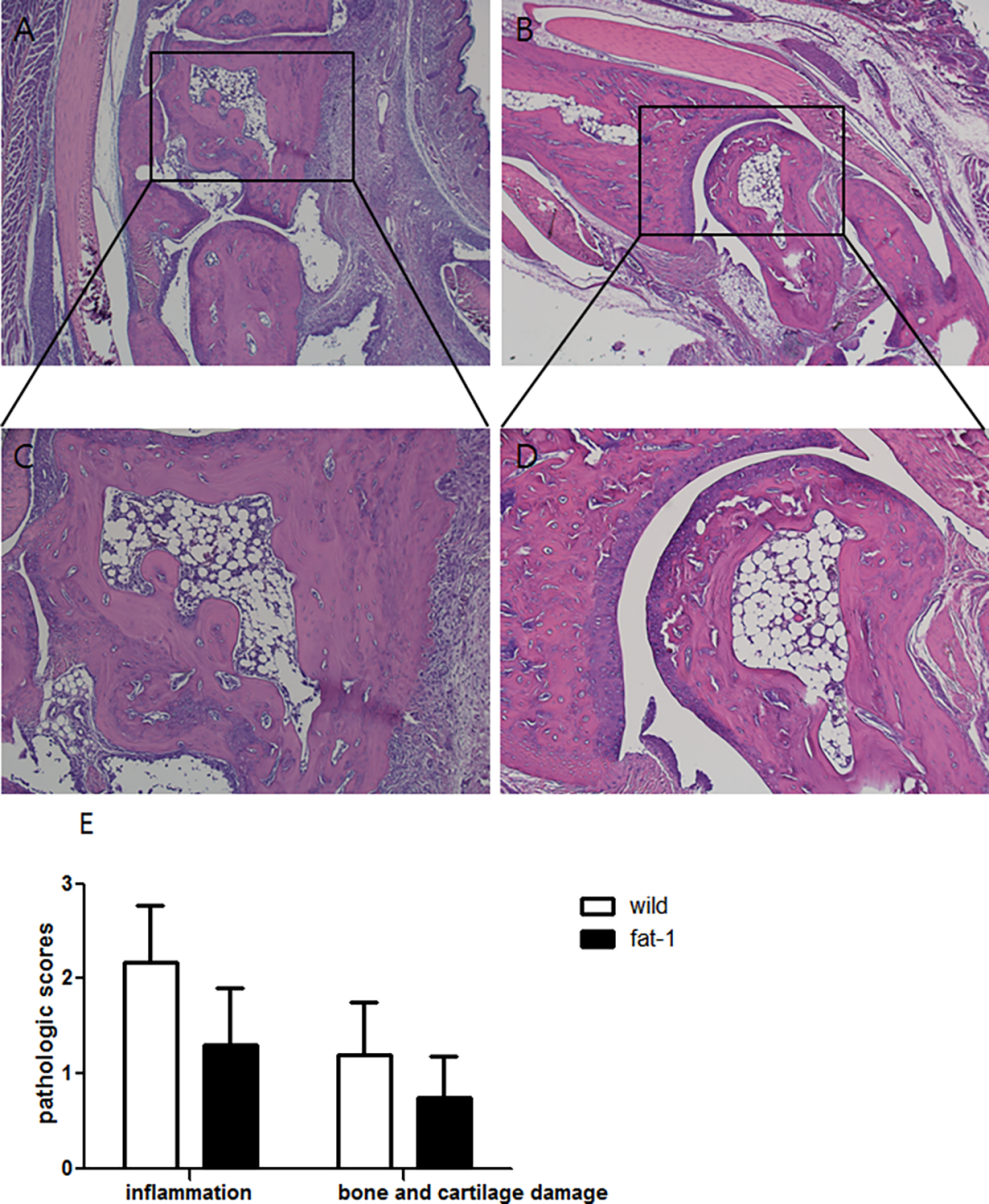
N-3 PUFAs inhibits ankle joint inflammation and bone and cartilage damage in CAIA mice. Ankle joints were collected on day 9, sectioned and stained with H&E in WT (A) and fat-1 mice (B). Photomicrographs (x40) are representative of at least three independent experiments. Further magnification of black-bordered box (x100) showed the typical inflammatory injuries in WT (C) and fat-1 mice (D). (E) Pathologic scores in the joint tissues of both groups were determined as described in the Materials and Methods. Values are the mean±SD (n = 5 mice/group).

Clinical arthritis score was significantly lower in fat-1 mice than in WT at both day 7 (1.6±1.8, p = 0.012) and day 9 (1.5±1.6, p = 0.003). Ankle thickness was also significantly reduced in fat-1 mice compared to WT mice (1.82±0.11, p = 0.008) at day 9. Next, we conducted histologic evaluation in the joints of mice. The pathological assessment showed that histological score of joint tissue regarding inflammation, bone and cartilage damage decreased in fat-1 mice compared to WT (Fig 2).

### N-3 PUFAs inhibits the production of inflammatory cytokines in joint tissues

**Fig 3:**
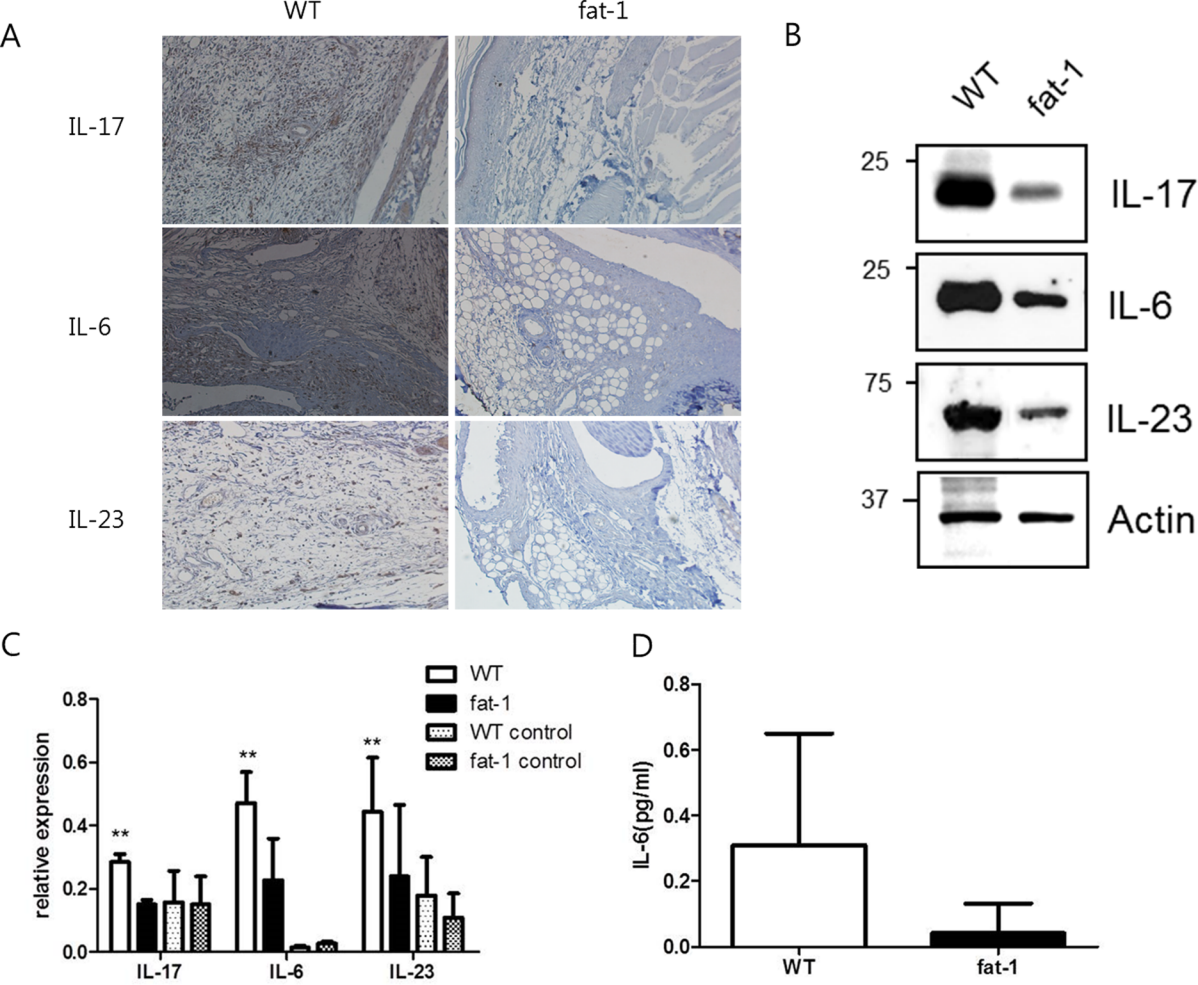
Pro-inflammatory cytokine production in the joint, spleen and serum of fat-1 and WT mice. Histological features of joint tissue were evaluated in the WT and fat-1 mice. Immunohistochemical staining (A) of cytokines was conducted in joint sections from WT and fat-1 mice. Ankle joints were collected on day 9, sectioned and stained with anti-IL-6, IL-23 and IL-17 antibodies. Western blots were conducted in the joint lysates from the WT and fat-1 mice (B). The joint lysates were collected on day 9 and blotted with anti-IL-6, IL-23 and IL-17 antibodies. The spleens were collected from WT and fat-1 mice on day 0 and 9. The expression of various pro-inflammatory cytokine in the spleen were measured by real-time Q-PCR (C). The serum were collected from WT and fat-1 mice on day 9. The expression of various pro-inflammatory cytokine were measured by ELISA (D). The results of three independent experiments with 5 mice/group are expressed as mean±SD. (*p<0.05, **p<0.01) Statistical significance test was done by Mann-Whitney U-test. WT: wild type, IL-7: interleukin 17, IL-6: interleukin 6, IL-23: interleukin 23.

Infiltration of inflammatory cells into joint tissues causes chronic inflammation and bone destruction in RA. IL-17 plays a critical role in the development of chronic inflammation leading to joint damage in RA [20]. Therefore, the expression of inflammatory cytokines in joint tissues was investigated in the WT and fat-1 mice. In the sections of joint tissues from WT mice, a large number of synovial cells expressed IL-17, IL-6 and IL-23. However, fat-1 mice showed that lower number of synovial cells expressed these cytokines compared to the WT mice (Fig 3A). Next, we conducted western blots on the joint lysates. The protein expression levels of IL-17 were significantly lower in fat-1 mice compared to WT mice (p = 0.029) (Fig 3B).

### The splenic expansion of inflammatory cytokines is attenuated in fat-1 mice

RA is a systemic autoimmune disease, and the spleen contains a variety of immune cells producing pro-inflammatory cytokines which play important roles in the development of chronic arthritis. Therefore, we investigated the expression level of various pro-inflammatory cytokines in the spleen of both groups. First, the mRNA of IL-17 was highly expressed in the spleen of WT mice. In contrast, the levels of IL-17 mRNA expression significantly decreased in the spleen of fat-1 mice (p = 0.032). In addition, the mRNA expression levels of IL-6 and IL-23 which are involved in the generation of IL-17 were significantly lower in the fat-1 mice than in WT (IL-6; p = 0.016, IL-23; p = 0.016). However, the expression level of TNF-α was comparable between the WT and fat-1 mice (Fig 3C). Next, we investigated those cytokines in the serum of both groups. Only IL-6 was expressed in the serum of the WT mice, and the level of which was lower in the fat-1 mice, but there was no significant difference between the WT and fat-1 mice (p = 0.190) (Fig 3D).

### The mRNA expression of Foxp3 in the spleen is elevated in the fat-1 mice

**Fig 4:**
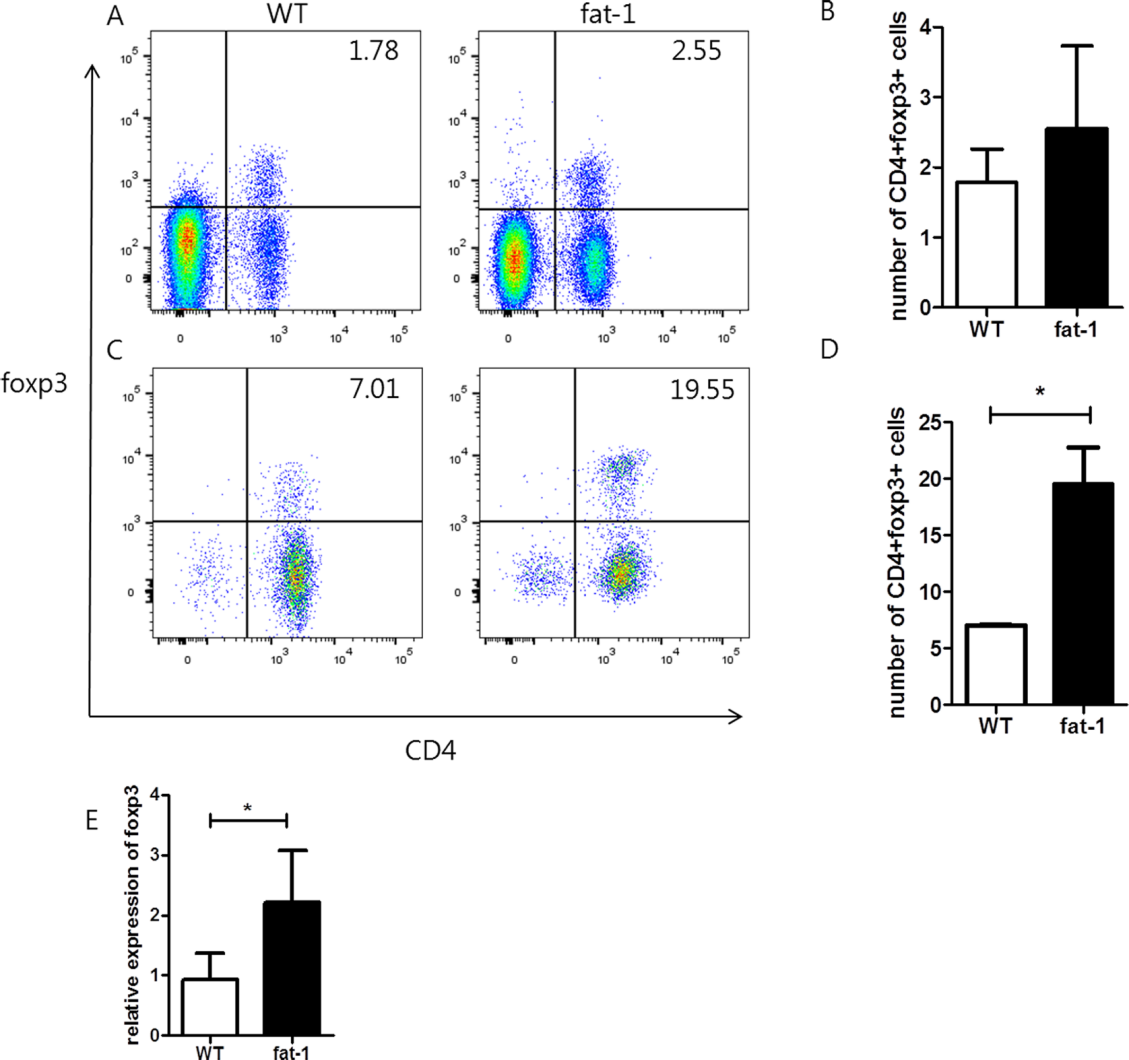
N-3 PUFAs induce the differentiation of CD4 T cells into splenic CD4^+^Foxp3^+^ regulatory T cells. The splenocytes of the WT and fat-1 mice were isolated, stained, and sorted by flow cytometry using anti-CD4 and FoxP3 antibodies (A and B). The percentage of double positive on the CD4 and Foxp3 in the WT and fat-1 are shown in A and B. The mRNA expressions of Foxp3 were measured by real-time Q-PCR (E).Treg differentiation was higher in fat-1 splneocytes than in the WT mice (C and D). CD4^+^ T cells were cultured with anti-TGF-β antibody for Treg differentiation. Values are the mean±SD (n = 5 mice/group). Statistical significance test was done by Mann-Whitney U-test. WT: wild type.

We obtained the spleen on day 9, and analyzed the proportion of Treg cells. The number of CD4^+^Foxp3^+^Treg cells in the spleen was determined using flow cytometry. The number of CD4^+^Foxp3^+^Treg cells tended to be higher in the fat-1 mice compared to WT mice (Fig 4A and 4B). But there was no significant difference (p = 0.264). Next, the mRNA expression of Foxp3 was measured by real-time Q-PCR. The mRNA expression level of Foxp3 is significantly higher in the spleen of fat-1 mice compared to WT mice (p = 0.009) (Fig 4E).

### The differentiation of CD4^+^ T cells into CD4^+^CD25^+^Foxp3^+^ Treg cells in the fat-1 and WT mice

Treg cells have been studied as controllers of immune response to prevent the development of chronic inflammatory diseases. Therefore, we analyzed whether administration of n-3 PUFA induced the generation of Treg cells. WT and fat-1 mice were sacrificed without the induction of CAIA. The splenocytes from each group were stimulated with anti CD3, CD28 and TGF-β antibodies to see if there were any differences in the development of Treg cells. The number of CD4^+^Foxp3^+^Treg cells in fat-1 splenocytes was significantly higher than that in WT splenocytes (p = 0.038). Therefore, n-3 PUFA affected the development of CD4^+^Foxp3^+^Treg cells in the spleen (Fig 4C and 4D).

## Discussion

The inflammatory cytokines expressed highly in RA joint enhance infiltration of inflammatory cells into RA joints culminating in joint tissue damage and bone destruction [21]. There are many reports that n-3 PUFAs improve inflammatory diseases such as panceratitis [16], diabetes [22] and asthma [17] by increase of anti-inflammatory effects. N-3 PUFAs are primarily obtained from diet. The dietary intake of fish oil has been considered beneficial in RA patient. Kromann N et al. reported intake of large amounts of fish oil decreased incidence of RA in Eskimos [23]. In animal study conducted in collagen-induced arthritis (CIA) model, prophylactic treatment with DHA significantly reduced arthritis severity and joint damage [24]. In a clinical trial, RA patients treated with n-3 PUFA showed reduced treatment failure and increased achievement of disease improvement [25]. In addition, it has been shown that n-3 PUFAs down-regulate pro-inflammatory cytokines and up-regulated anti-inflammatory mediators in RA [17, 26]. However, other studies reported controversial results [27, 28]. Therefore, the efficacy of n-3 PUFA on RA has not been clarified yet.

In this study, we verified whether n-3 PUFAs attenuated chronic autoimmune arthritis by using fat-1 mice which contain the fat-1 gene from *Caenorhabditis elegans* and synthesize n-3 PUFAs through converting n-6 into n-3 PUFA. The advantage of using fat-1 mice is to eliminate confounding factors of diet leading to providing reliable evidence. The representative animal model for the study of RA is the CIA mice. However, CIA is induced in mice with DBA/1 background rather than in those with a C57Bl/6 genetic background including the fat-1 mice. Therefore, we used the collagen antibody-induced arthritis model to induce arthritis in the fat-1 mice.

In our study, clinical phenotypes were generally improved in the fat-1 mice in which n-3 PUFAs decreased inflammation in the CAIA model. To find out the mechanisms by which n-3 PUFAs reduce inflammation in the joint tissues, we investigated cytokine profile. CD4 T cells are major population in synovial tissues and IL-17 has been shown in high level in synovial fluid of RA patients [29]. Since Th17 cells produce pro-inflammatory cytokine IL-17, Th17 cells have been suggested to play a critical role in the pathogenesis of RA. Th17 cells are differentiated from naïve CD4+ T cells. IL-6 and TGF-β promote Th17 differentiation, and IL-23 preserves the Th17 cell functional phenotypes [2]. On the other hand, Treg cells suppressed inflammatory signals by secretion of anti-inflammatory cytokines, such as TGF-β and IL-10 [30]. Treg cells are developed in either the thymus or lymphoid tissues such as spleen, lymph node and intestinal mucosa. Treg cells require CD28 co-stimulation during positive selection in the thymus in the presence of TGF-β, IL-2, and IL-15 [31]. In patient with RA, Treg cells are functionally defective, and restoring Treg cells function is important for the control of inflammation and restoring tolerance in RA patients [32].

Recently, N-3 PUFAs have been reported to increase the number of CD4+CD25+Foxp3+Treg cells[11, 33–36] and to decrease Th17 cell differentiation and IL-17 production in other disease models including encephalomyelitis and psoriasis [36–38]. In our study, n-3 PUFAs significantly decreased IL-6, IL-23 and IL-17 expression while increased the number of Treg cells in the joint tissues of the fat-1 mice. The spleen plays an important role in regulating the immune system and contains a variety of immune cells. The expression of IL-6, IL-23 and IL-17 was decreased while the expression of foxp3 was increased in the spleen of the fat-1 mice. The number of Treg cells was similar between the fat-1 and WT mice, but after stimulation, the differentiation to Treg cells was significantly higher in the fat-1 mice than in the WT. Therefore, our results suggest that anti-inflammatory function of n-3 PUFAs does not restrict to joint tissue but also works in the immune organs. However, in the serum, we could find only IL-6 and the level of which was not significantly different between the fat-1 and WT mice. This may account for the development of milder arthritis in C57Bl/6 strain induced by anti-collagen antibodies than in DBA/1 strain of the CIA model.

**Fig 5:**
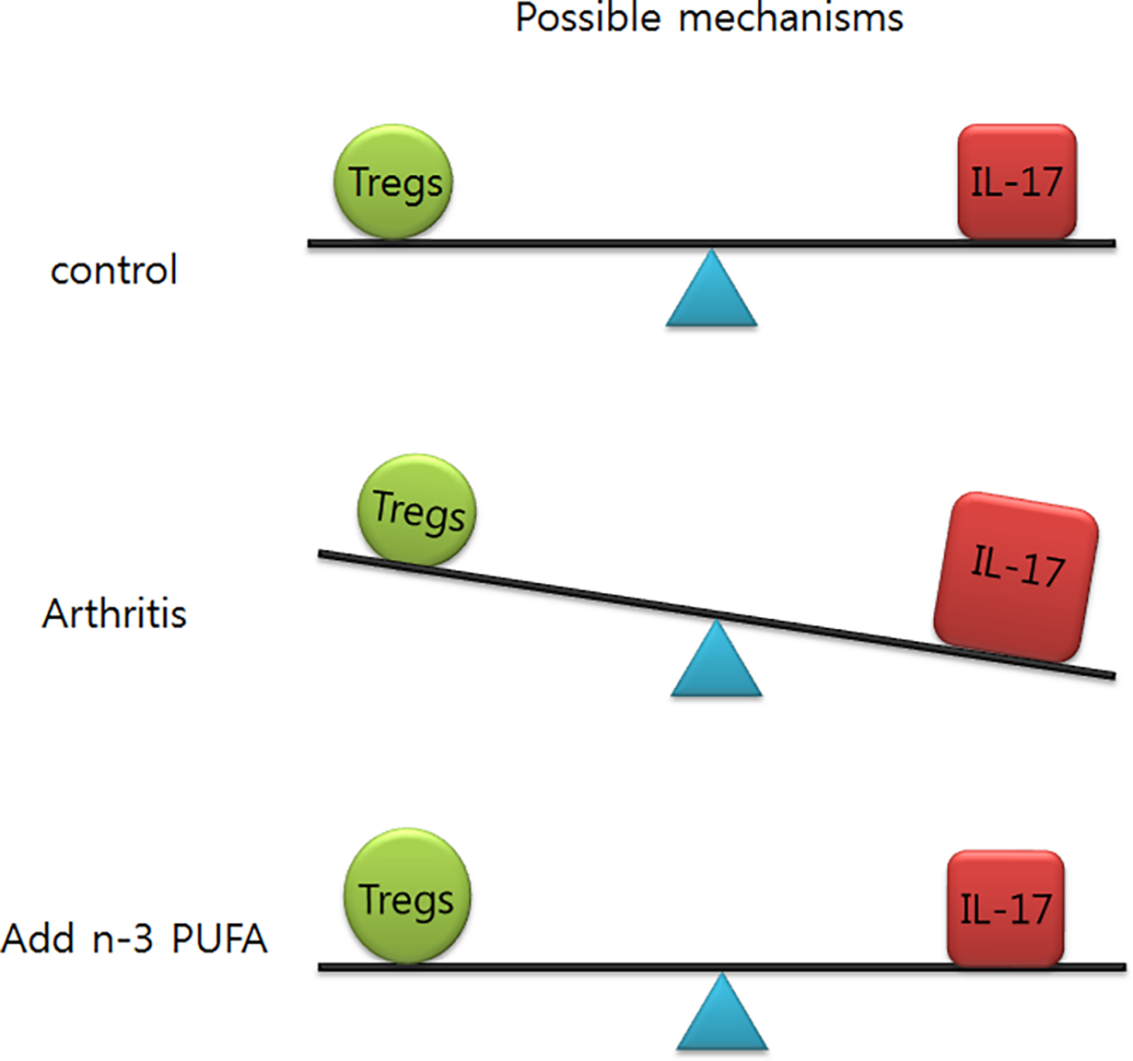
Hypothetical mechanism by which n-3 PUFAs reduce the severity of arthritis in RA model. Imbalance of Th17/Treg cells in the CAIA model could be restored by n-3 PUFA.

Previous studies have reported that the imbalance of Th17/Treg cells in the peripheral blood, may play an important role in RA development [39–41]. Therefore, our results suggest that n-3 PUFAs could improve arthritis through restoring Th17/Treg cell balance (Fig 5). The restoring Th17/Treg cell balance could be a therapeutic strategies in RA patient such as type II collagen induces peripheral tolerance through the generation of Treg cell [42–43].

Similarly n-3 PUFA, granulocyte macrophage colony-stimulation factor (GM-CSF) has been shown to prevent or attenuate autoimmunity in a number of mouse models of autoimmune disease by expanding regulatory T cells [44–45]. Rowin J et al. reported that treatment with GM-CSF was associated with clinical improvement, an expansion of the circulating number of Foxp3+ cells [46]. The pro-inflammatory and regulatory effects of GM-CSF appear to depend on the dose and the presence of other relevant cytokines in the context of an immune response [47]. In contrast, a human monoclonal antibody against GM-CSF has been shown to have moderately positive benefits in patients with RA [48]. Based on these previous researches, GM-CSF might be has a synergic effect with n-3 PUFA and further study is needed. The result of in this study points to n-3 PUFA as potential therapeutic agents that contribute to improving the mechanisms that caused RA.

Some previous study reported that the affection of n-3 PUFA to M1 macrophage. In general, M1 macrophages are efficient producers of proinflammatory cytokines. Adipose tissue macrophage (ATM) is an anti-inflammatory phenotype and capable of excessive proinflammatory mediator production. Incorporation of n-3 PUFA in diet prevents mcrophage infilteration induced by high fat diet, which is similar with another research revealing reduction in ATM, along with transformation from M1 to M2 polarization stage [49].

On the side of osteoclastogenesis, dietary fish oil suppressed osteoclastogenesis by inhibiting the expression of M-CSF and RANK in the early stage of osteoclastogenesis [50], which is consistent with another research that DHA were the effective in inhibiting RANKL-induced osteoclast formation with the latter providing the strong inhibitory effects [51].

The anti-inflammatory effects of n-3PUFAs occur through multiple mechanisms. N-3PUFAs down regulated pro-inflammatory cytokine synthesis such as TNF-α, IL-1β, IL-6, IL-23 and IL-8 through decreased activation of nuclear transcription factors such as NF-kB [36, 37].

Our study had several limitations. First, we used collagen antibody-induced arthritis model which does not perfectly reflect the pathogenesis of human RA. Secondly, absence of severe inflammation in serum makes it difficult to find strong evidence of systemic immune response in C57Bl/6 strain. However, our CAIA model showed similar results with those found in the CIA model which depends on adaptive immune response.

In conclusion, we confirmed that n-3 PUFAs attenuated development of arthritis probably through inhibition of generation of IL-17 mediated by IL-6 and IL-23, and increased the expression of foxp3 in the fat-1 mice. These findings suggest that n-3 PUFAs might be a promising therapeutic agent for the treatment of RA.

## Supporting information

S1 Checklist The arrive Guidelines checklist. (DOCX)

S1 Data The morphology scoring result of fat-1 and wild mice. (XLSX)

S2 Data The pathology reading result of fat-1 and wild mice in day 9. (XLSX)

S3 Data The RT-PCR result of inflammatory cytokines. (XLSX)
