# Supplementary material for: N-3 polyunsaturated fatty acids restore Th17 and Treg balance in collagen antibody-induced arthritis
Source: PLoS One. 2018 Mar 15;13(3):e0194331. doi: 10.1371/journal.pone.0194331 (PMC5854360; doi:10.1371/journal.pone.0194331)

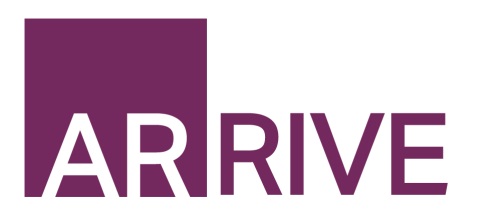


The ARRIVE Guidelines Checklist

Animal Research: Reporting In Vivo Experiments

Carol Kilkenny^1^, William J Browne^2^, Innes C Cuthill^3^, Michael Emerson^4^ and Douglas G Altman^5^

*^1^The National Centre for the Replacement, Refinement and Reduction of Animals in Research, London, UK, ^2^School of Veterinary Science, University of Bristol, Bristol, UK, ^3^School of Biological Sciences, University of Bristol, Bristol, UK, ^4^National Heart and Lung Institute, Imperial College London, UK, ^5^Centre for Statistics in Medicine, University of Oxford, Oxford, UK.*

|  | | ITEM | RECOMMENDATION | Section/ Paragraph |
| --- | --- | --- | --- | --- |
| 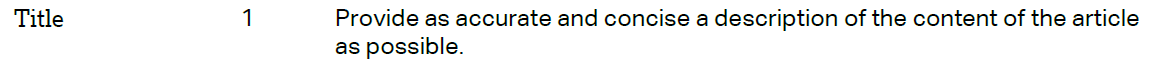 | | | Title |  |
| 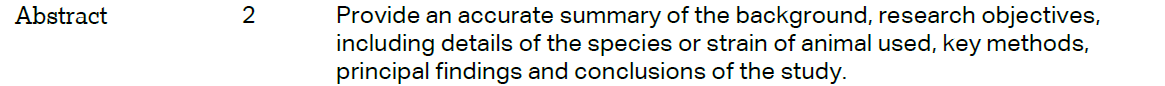 | | | Abstract |  |
| INTRODUCTION | | |  |  |
| 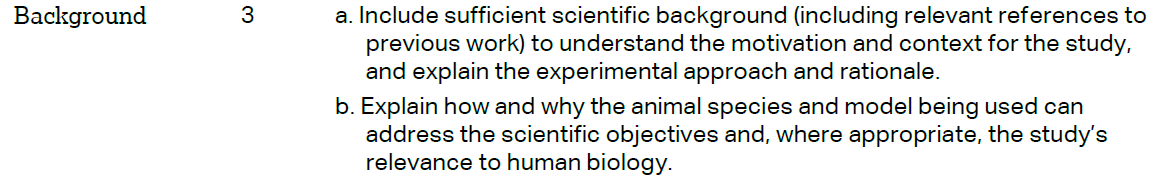 | | | Paragraph 1-2  Paragraph 3 |  |
| 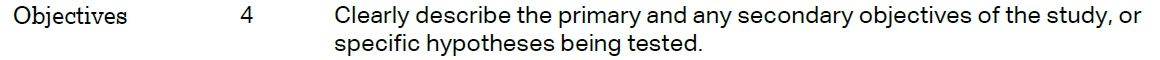 | | | Paragraph 4 |  |
| METHODS | | |  |  |
| 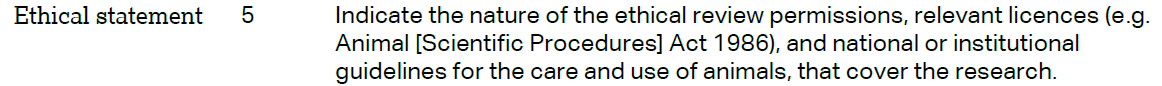 | | | Paragraph 1 |  |
| 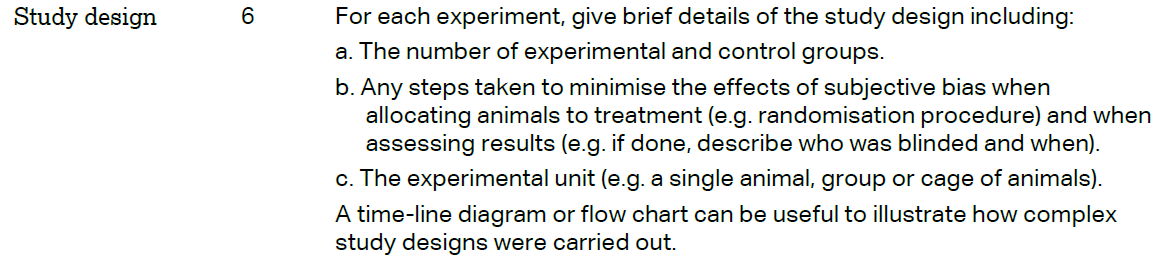 | | | Paragraph 1 |  |
| 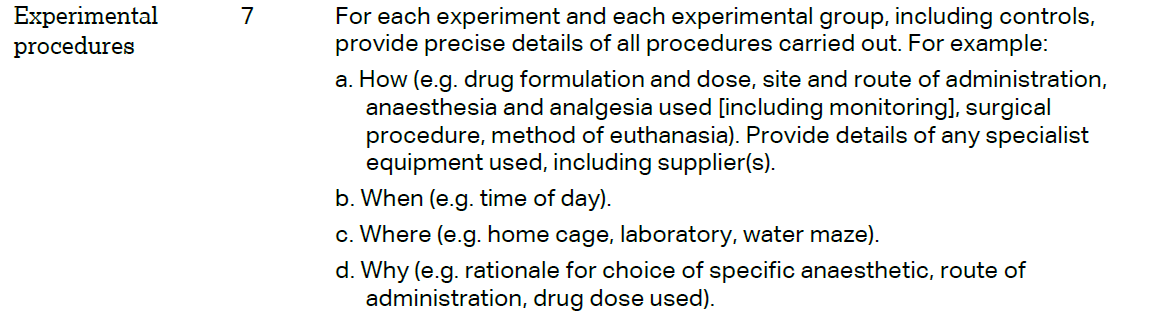 | | | Paragraph 2-4 |  |
| 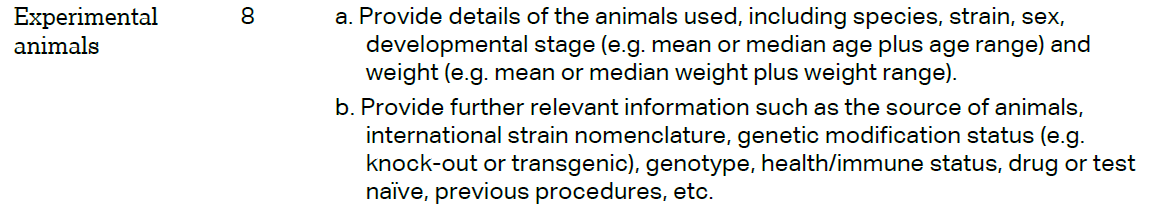 | | | Paragraph 2 |  |

The ARRIVE guidelines. Originally published in *PLoS Biology*, June 2010^1^

| 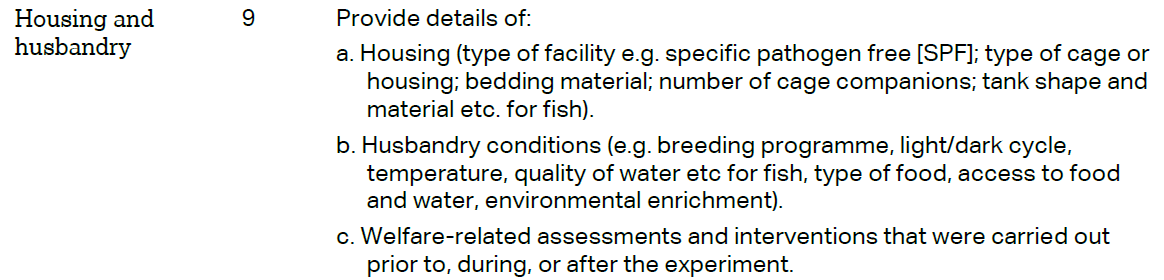 | Paragraph 1 | |
| --- | --- | --- |
| 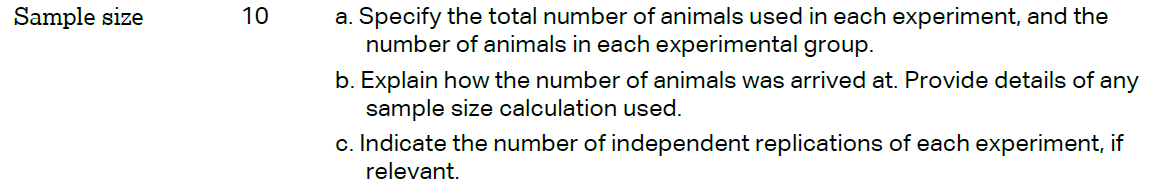 | Paragraph 1 | |
| 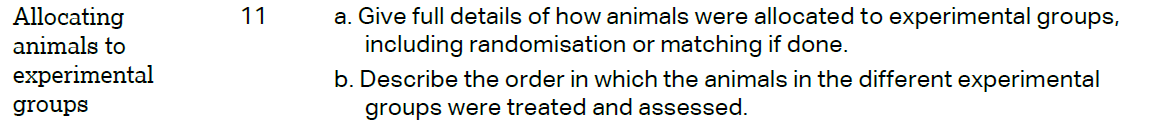 | Paragraph 4 | |
| 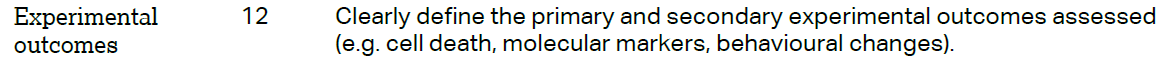 | Paragraph 4 | |
| 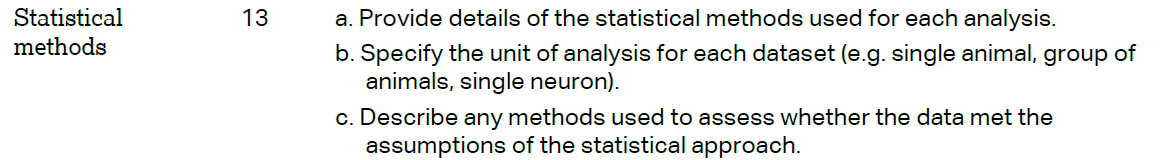 | Paragraph 13 | |
| RESULTS |  | |
| 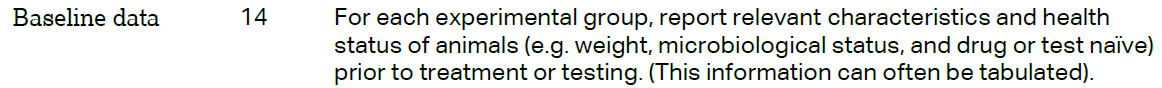 | Paragraph 1 and Figure 1 & 2 | |
| 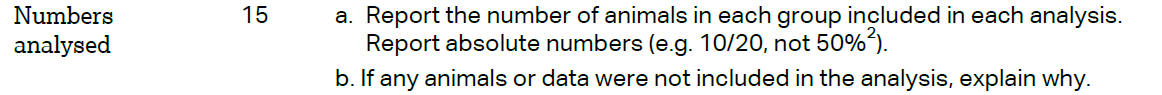 | Method Paragraph 1 | |
| 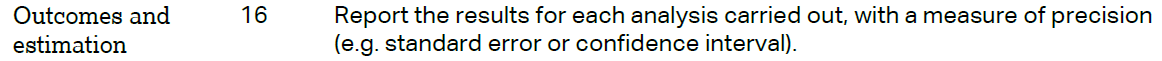 | Paragraph 1-6 and Figure 3&4 | |
| 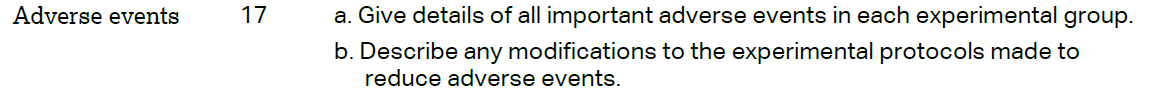 | Paragraph 1 | |
| DISCUSSION |  | |
| 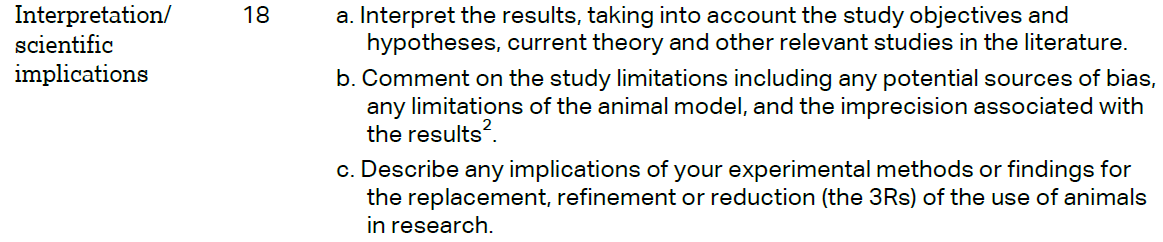 | Paragraph 1-8,  Paragraph 10  Paragraph 2 | |
| 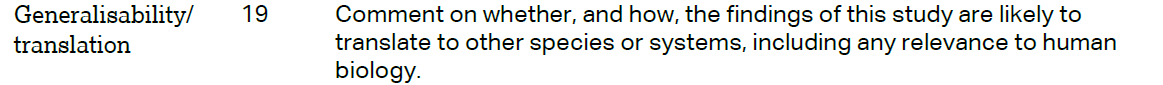 | Paragraph 6 | |
| 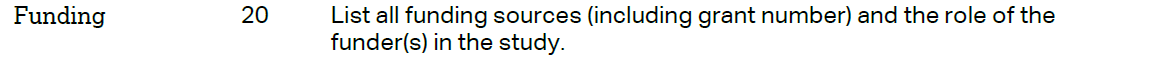 | | Acknowledgement paragraph 1 |


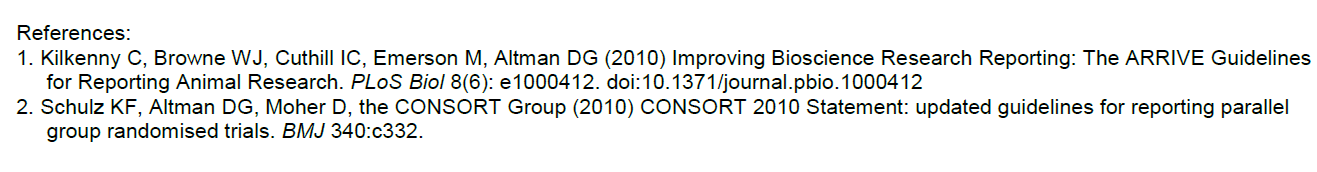

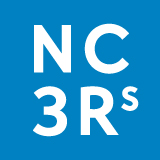

Supplement: S1 Checklist — (DOCX) [file pone.0194331.s001.docx]
